# Effects of Salt-Drought Stress on Growth and Physiobiochemical Characteristics of *Tamarix chinensis* Seedlings

**DOI:** 10.1155/2014/765840

**Published:** 2014-07-22

**Authors:** Junhua Liu, Jiangbao Xia, Yanming Fang, Tian Li, Jingtao Liu

**Affiliations:** ^1^College of Biological and Environmental Sciences, Nanjing Forest University, Nanjing 210037, China; ^2^Shandong Provincial Key Laboratory of Eco-Environmental Science for Yellow River Delta, Binzhou University, Binzhou 256603, China

## Abstract

The present study was designed to clarify the effects of salinity and water intercross stresses on the growth and physiobiochemical characteristics of *Tamarix chinensis* seedlings by pots culture under the artificial simulated conditions. The growth, activities of SOD, POD, and contents of MDA and osmotic adjusting substances of three years old seedlings of *T. chinensis* were studied under different salt-drought intercross stress. Results showed that the influence of salt stress on growth was greater than drought stress, the oxidation resistance of SOD and POD weakened gradually with salt and drought stresses intensified, and the content of MDA was higher under severe drought and mild and moderate salt stresses. The proline contents increased with the stress intensified but only significantly higher than control under the intercross stresses of severe salt-severe drought. It implied that *T. chinensis* could improve its stress resistance by adjusted self-growth and physiobiochemical characteristics, and the intercross compatibility of *T. chinensis* to salt and drought stresses can enhance the salt resistance under appropriate drought stress, but the dominant factors influencing the physiological biochemical characteristics of *T. chinensis* were various with the changing of salt-drought intercross stresses gradients.

## 1. Introduction

*Tamarix chinensis* belongs to the Tamarix family (Tamaricaceae), which are mainly xerophytes. It is native to China and Korea, and it is known in many other parts of the world as an introduced species and sometimes an invasive noxious weed. It easily inhabits moist habitat with saline soils.* T. chinensis* as an ornamental plant was introduced into North America at the beginning of nineteenth Century and later into the southwest American desert regions for windbreak and sand fixation.* Tamarix chinensis* receives more attention amongst different Tamarix species [1]. Because of its strong adaptability to the local environment,* T. chinensis* replaced the native species, and as the rapid invasion to many rivers and desert edges results in the loss of biodiversity in arid area, water consumption increased and a series of related problems are listed as USA ten alien invasive species [2, 3]. As a native species in China,* T. chinensis* is mainly distributed in the northwest arid area of Xinjiang, Inner Mongolia and Gansu, and it is also the main species for vegetation restoration of ecological restoration and protection for the coastal wetlands of Bohai Gulf. The Yellow River Delta is the most concentrated distribution area of* T. chinensis* [4], but this area is being threatened by hydrological changes [1]. In recent years, due to the large amount of groundwater exploitation of local area, seawater intrusion led to soil salinity and the evaporation to precipitation ratio increased. Due to the lack of freshwater resources, the salt and drought stress became the two major effect factors on local* T. chinensis* seedlings growth [4, 5].

As known, soil is an essential component of wetland ecosystem, which can support, hold, and regulate water and nutrients [6]. However, the effects of drought stress and salt stress on the growth and development of plants are coupled together, by reducing the soil solution water potential. Many of the previous studies are focused on the interaction between salt and drought and its effect on plant growth and physiological and biochemical characteristics [7–10]. The effects of salt and drought stress on plant physiological and biochemical properties are mainly concentrated in the* Ammodendron bifolium* [7, 10],* Gleditsia sinensis* [8], and* Cercis chinensis Bunge* seedlings [9]. But the stress physiology researches of* T. chinensis* mainly concentrated on single salt stress [11–14] or drought stress [15, 16], such as salt stress on the growth of* T. chinensis* [11], physiological and biochemical characteristics during the leaf expansion period [17], photosynthesis and osmotic adjustment substances [12], and salt secretion characteristics and influence factor under saline habitats [13]. Furthermore, the drought resistance mechanism under different stress levels [15] and physiological and ecological characteristics [16] under different environment had been carried out. But the physiological and biochemical characteristics response of* T. chinensis* to the salt-drought intercross stress conditions has not been reported. This study was aimed at analyzing the change dynamic of biomass, superoxide dismutase (SOD), peroxidase (POD), malondialdehyde (MDA), and osmotic adjusting substances under different gradients of salinity stress-drought intercross stresses treatment, simulating the soil moisture and soil salinity conditions of sea-side and land-side and clarifying the growth and physiobiochemical characteristics of* T. chinensis* under different salt-drought stresses conditions.

## 2. Materials and Methods

### 2.1. Plants

Three-year-old* Tamarix chinensis* seedlings were selected from National Marine Ecological Conservation Region at Changyi county in Shandong Province, China. Previous works indicated that these seedlings had homogenesis traits in physiological and morphological aspects.

### 2.2. Experimental Setup

The experiments were carried out in a greenhouse at Binzhou University, Binzhou, China, from March 21, 2013, to June 15, 2013.* T. chinensis *seedlings of 3a old had been transplanted to research greenhouse, cocultivated using pots diameter 30 cm, high 50 cm, 1 plants per pot, a total of 24 plants, potted matrix for sandy loam soil (salt content 0.02%).* T. chinensis* seedlings normal growth after April 20th 2013 to salt and drought stress treatment. Drought stress treatment using Hsiao [14] moisture gradient design method, includes high soil moisture (mild drought stress) and low soil moisture (serve drought stress); the soil relative water content was 55%~60% and 30%~35%. Salinity (soil salinity/soil dry weight) by NaCl solution configuration of different gradient times irrigation to control, set up 2 levels, the salt concentrations were 0.4%, 1.2% and 2.5%, and the salt content of 0.02% as control. The pot bottom is provided with a tray whose depth is 8 cm, the leakage water into the pot and the cleaning tray, clean water is poured into the pot to prevent salt loss [18]. The experiment consists of 8 (2 × 4) salt-drought treatments. Each treatment was repeated 3 times and was designed by randomized block arrangement. Salt and drought stress treatment for 8 weeks (From April 20th to June 15th), determination of sample indexes.

### 2.3. Plant Sampling and Analysis

The same position of* T. chinensis* seedlings mature leaves was collected and dried, ground and sieved through a 120 mesh sieve, and sealed with plastic bags to be measured.

#### 2.3.1. Physiological and Biochemical Indexes

The SOD activity was determined by nitro blue tetrazolium [19, 20] four photochemical reduction method; the activity of POD [21] was measured using guaiacol colorimetric method; MDA was measured by TBA colorimetric method [19].

#### 2.3.2. Determination of Proline Content

Take 0.05 g of dry sample into a centrifuge tube, sulfosalicylic acid with 10 mL 3%, sealing bag seal affixed after boiling water bath extraction for 30 min; after cooling centrifugal (3000 r/min, 10 min), 1 mL supernatant fluid, 1 mL 3% sulfosalicylic acid, acetic acid, and 2 mL 2.5% acid indene ketone were added to three glass test tubes (tube with glass balls) in boiling water bath for 60 min chromogenic reaction; then we cool them to room temperature to join the 4 mL toluene and shake extracted red material, still taking the toluene layer, in the 520 nm absorbance (OD) [19].

### 2.4. Statistical Analysis

All statistical tests were performed using SPSS 18.0. Two-way ANOVA was used to determine the significance of different salt and drought treatment effects on growth and the physiological characteristics. Mean treatment differences were separated by the LSD test (*P* < 0.05 and *P* < 0.01) if *F*-tests were significant.

## 3. Results

### 3.1. Effect of Salt and Drought Stresses on the Growth Traits of* T. chinensis*

As can be seen from Table 1,* T. chinensis* plant height increased more significantly (*P* < 0.05) in the salt content at 0.4% level, respectively, 34.7% and 15.0%, than CK under high and low soil moisture, but the plants died under the stress condition with salinity 2.5% and high soil moisture (see Figure 2). The main root length of* T. chinensis* showed no significant difference (*P* > 0.05) between 0.4% salt stress treatment and CK under high soil moisture level, but the main root length of 1.2% salt stress treatment was shorter obviously than CK (*P* < 0.05). Under low soil moisture condition, the main root length increased first and then decreased, and the main root length of 1.2% salt stress treatment was significantly longer than CK (*P* < 0.05). The basal diameter of* T. chinensis* increased first and then decreased with the increase of the salt content in the two kinds of soil moisture conditions and reached the maximum at 0.4%, and the salt content of 1.2% was significantly decreased (*P* < 0.05).

Under the mild and severe drought stress, aboveground parts and underground parts dry weight of* T. chinensis *was both increased first and then decreased with the salt stress increase, and reached the maximum value at the salt content of 0.4%. In addition, plant individuals of* T. chinensis* still alive under the severe drought stress, while died in mild drought under severe salt stress, it may be appropriate drought stress can improved* T. chinensis *to a certain extent of salt tolerance to water logging and saline habitats. The above results showed that the effects of salt stress on the growth of* T. chinensis*  were more than drought stress, although there are certain inhibitory effects of drought stress on* T. chinensis*. In the meanwhile, the variation degree of aboveground dry weight was higher than those of the underground parts under the drought stress and salt stress treatments.

### 3.2. Effects of Salt and Drought Stresses on SOD and POD Enzymes Activities of* T. chinensis* Seedlings

**Figure 1:**
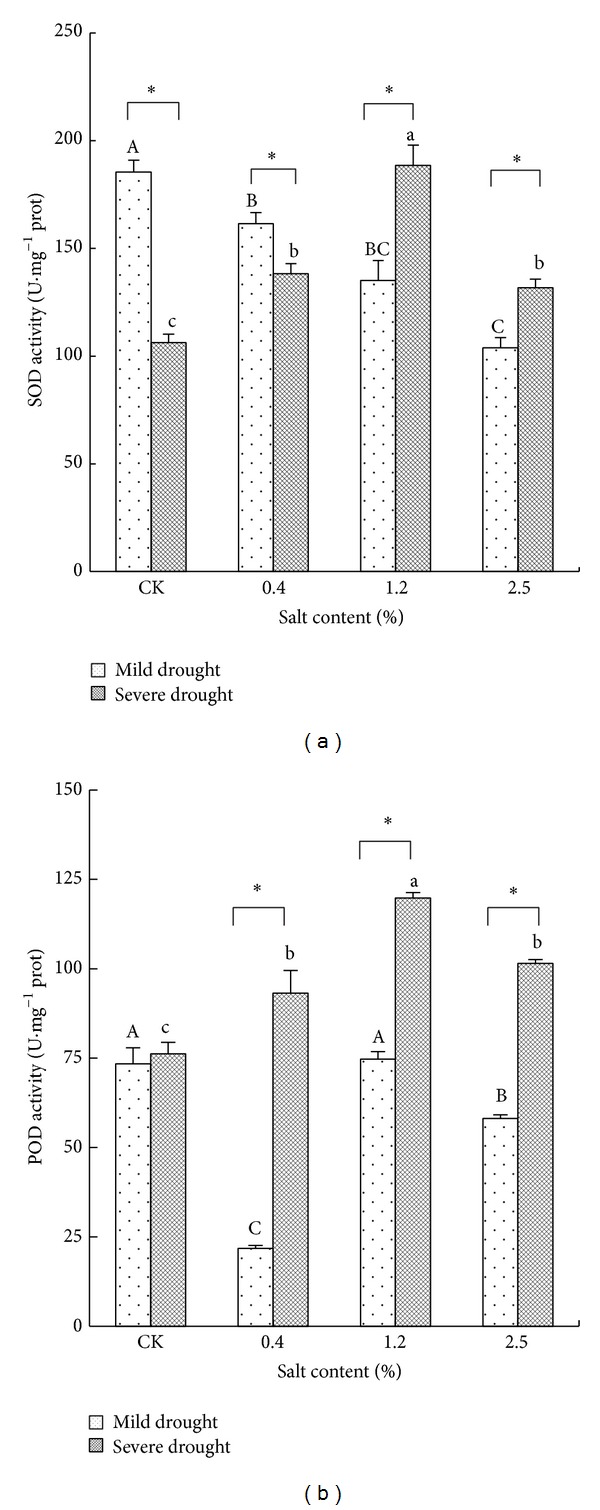
The changes of SOD and POD activity under different salt and drought stresses. The different letters within the same treatment indicate the significant difference at 0.05 level. The “∗” stands for the significant difference at 0.01 level between two treatments under the same salt stress.

**Figure 2:**
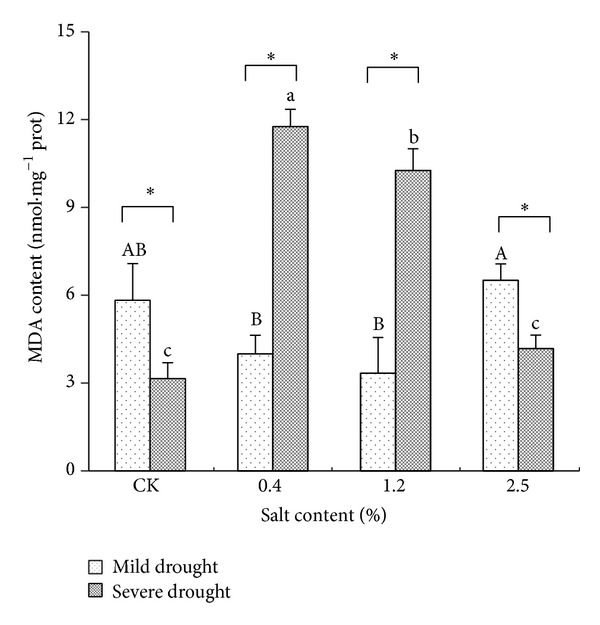
The changes of MDA contents under different salt and drought stresses. The different letters within the same treatment indicate the significant difference at 0.05 level. The “∗” stands for the significant difference at 0.01 level between two treatment under the same salt stress.

We can see from Figure 1, under mild drought stress, SOD activity in leaves of* T. chinensis *increased with the salt content decrease, and the difference between the salt stress treatment was significant (*P* < 0.05); compared with CK, SOD activity of leaves decreased significantly in the salt content of 0.4% (*P* < 0.05) and decreased obviously in the salt content of 1.2% and 2.5% (*P* < 0.01). Under low soil moisture conditions, the SOD activity in leaves increased first and then decreased with the salinity increasing, and the activity of SOD increased about 30% and 80%, respectively, compared with CK in the salinity of 0.4% and 1.2%, then reached the maximum value at salinity 1.2%, and was significantly lower in the salt content of 2.5% (*P* < 0.05). Under the same salt stress, the effects of different drought stress and salt stress effect on SOD activity were significant (*P* < 0.01). By the double factor variance analysis, SOD activity in leaves of* T. chinensis* by containing salt and salt drought intercross stress was significant (*P* < 0.01).

Under mild drought stress condition, the leaf POD activity decreased first and then increased and then decreased with the increase of soil salt content; the difference among all treatments was extremely significant (*P* < 0.01). Under severe drought stress, POD activity in leaves is increased first and then decreased with the increasing of salinity, and the salt content of 0.4% and 1.2% was increased 20% and 50%, respectively, more than those of CK and decreased approximately 15% compared to those in the salt content 2.5% (*P* < 0.05). Under the same salt stress, the effects of different drought stress and salt effect on POD activity also reached extremely significant level. Double factor variance analysis showed that salt stress, drought stress, and salt drought intercross stress had significant effects on POD activity of* T. chinensis* leaf.

### 3.3. Effects of Salt and Drought Stresses on MDA Content

MDA content in leaves of* T. chinensis* was firstly decreased and then increased under mild drought stress and reached the minimum value in the salt content of 1.2% and 0.4%. Under severe drought stress, the content of MDA in leaves increased first and then decreased and reached the maximum (11.96 nmol *·* mg^−1^ prot) at salinity 0.4%, 3.75 times of CK (*P* < 0.01). The content of MDA in leaves decreased in the salinity 1.2% but is still significantly higher than that of control. At the salinity of 2.5%, MDA content significantly decreased compared to other treatments but had no significant difference compared to the control. Double factor variance analysis showed that the effect of salt stress on the content of MDA was significant (*P* < 0.05), and drought stress or salt drought intercross stress effect on the MDA content reached extremely significant level (*P* < 0.01). Under the severe drought and light salt stress, the content of MDA reached a maximum value.

### 3.4. Effect of Salt Stress and Drought Stress on Proline Content in Leaves

**Figure 3:**
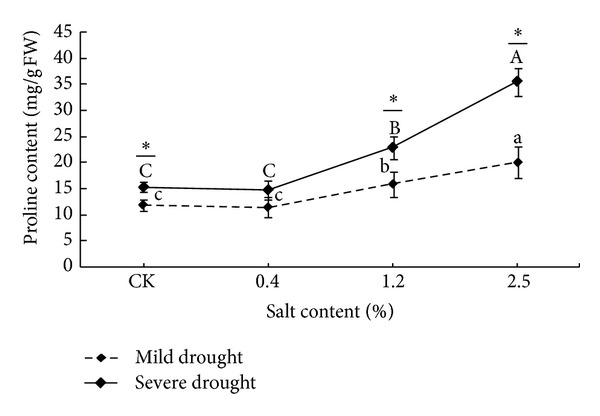
The changes of proline contents under different salt and drought stresses. The different letters within the same treatment indicate the significant difference at 0.05 level. The “∗” stands forthe significant difference at 0.01 level between two treatments under the same salt stress.

As seen from Figure 3, the proline content in the leaves of* T. chinensis* increased at different extent with the increase of salt and drought stress in this study.

Under light drought stress condition, the proline content increased gradually with salt stress increases, but the had no significant difference compared with CK (*P* > 0.05); under the severe drought, the proline content in leaves decreased first and then increased with the enhancement of salt stress intensity, and the content at the salinity of 2.5% was significantly higher than that of CK (*P* < 0.05). When the salt concentration was below 2.5%, the proline content under different drought stress had no significant difference (*P* > 0.05). Double factor variance analysis showed that both salt stress and drought stress had significant effects on the content of proline in leaves of* Tamarix* seedlings (*P* < 0.05), and the mild drought stress is able to enhance accumulation of proline in leaves of* T. chinensis*, to adapt to the saline environment.

## 4. Discussion

### 4.1. Salt-Drought Intercross Stress Affected Plant Growth and Biomass Allocation

As usually, salt-drought stress can delay plants growth and inhibit differentiation of plant tissue and reduce fresh weight of leaf, stem, and root with the salt stress increase [22]. Previous study showed that soils along the coastal area could form gradients of salinity and vegetation development during the desalinization process [23]. With the salt content increasing,* Tamarix hispida *stress symptoms become more obvious, survival rate decreased, and high growth was inhibited [11].* Xanthoceras sorbifolia* seedlings adapt to drought stress by adjusting the biomass allocation and modified shape to achieve efficient use of existing habitat resources [24]. This study showed that* T. chinensis* had stronger tolerance of drought stress, but its growth was affected significantly by salt stress. Furthermore, the changing of* T. chinensis* biomass is obvious with the salt stress intensified and to adapt to high sanity environment by reducing plant height, basal diameter, and dry matter weight. In a word,* T. chinensis* maintained the normal growth under salt and drought conditions by adjusting the biomass allocation and its form.

### 4.2. Salt-Drought Stress Affected Enzyme System

SOD and POD are the main antioxidant enzymes in plants [7–9, 16] and play an important role in scavenging superoxide ion, resisting lipid peroxidation, reducing membrane damage, and so forth. The previous research results showed that, under stress conditions, the intensity and rise or fall of drought resistance related enzymes activity were related closely to plant species or varieties [25]. During experiment progress, enzyme activity increased with the stress increasing or first increased and then decreased. Like* Gleditsia sinensis* Lam, its SOD and POD activity first increased and then decreased under salt drought intercross stress condition, and in the same treatment condition, both SOD and POD activities were decreased along with the extension of treatment time. This study showed that leaf SOD and POD activity of* T. chinensis* had different change patterns under different intercross salt and drought stress. Under light drought stress, SOD activity decreased; POD activity decreased first and then increased and then decreased; this dynamic progress was related to that the low concentration of salt stress is able to alleviate the effects related to drought stress. In contrast, under severe salt stress, protective enzyme system was breached and enzyme activity was inhibited strongly, leading to further reduce in the moderate activity. Under the severe drought condition, the activities of SOD and POD decreased so obviously that it was not enough to clear free radicals in the body; then it resulted in lipid peroxidation and the damage of membrane system [8, 16].

### 4.3. Hydraulic-Salt Stress Affected MDA Contents

MDA is often used as a major index to judge the membrane lipid peroxidation, and its content represents the degree of damage [7, 8, 26]. Researchers found that MDA content of* Ammodendron bifolium *and* Gleditsia sinensis *seedlings membrane permeability increased along with the salt and drought intercross stress intensified. This study showed that appropriate salt drought intercross stress can weaken the peroxidation of membrane lipid, and then MDA content is relatively low, with less damage to the membrane system. However, under severe drought and mild to moderate salt stress,* T. chinensis* accumulated so much free radical that initiated peroxidation of membrane lipid and then injured cell membrane. But in severe salt and drought stress, MDA content decreased significantly, which may be a dominant factor and its salt drought intercross stress adaptive regulation; its mechanism still needs further analysis and discussion.

### 4.4. Salt-Drought Stress Affected Osmotic Adjusting Substances Contents

Osmotic adjusting substances play an important role for plants during the progress of growth, development, and reproduction in salt and drought stress [27]. Under natural conditions, plants frequently suffer multiple environmental stresses, and the combined effects of environmental stresses on plants are complex and are not equal to the sum by simply adding the effect of each single factor [28]. Under the salt and drought stress conditions, large accumulation of intracellular soluble sugar and proline improves the cell sap concentration, maintains normal cell turgor, prevents excessive water loss, and enhances the resistance of plants [29]. A large number of studies showed that the soluble sugar is one kind of important organic osmotic regulators under adversity condition; it not only has a stabilizing effect on cell membrane and protoplast and providing carbon skeletons and energy for the synthesis of protein but also indirectly transmuted into proline [30]. This study showed that, in the salt drought intercross stress, soluble sugar contents in the leaves of* T. chinensis* seedlings gradually increased with the stress degree enhancement, and in severe salt and drought stress, the soluble sugar content began to decrease. That is to say that the soluble sugar plays an important role in osmotic adjustment, but the permeability of soluble sugar regulation has certain limitations; for example, under serve drought stress, the osmotic adjustment ability of* T. chinensis *reduced greatly or was lost. Proline is usually considered as one of main osmotic substances for plants used to regulate potential balance of cytoplasmic and vacuolar infiltration under salt stress [31]. This study showed that proline of* T. chinensis* increased gradually with increasing of salt and drought stress, but under mild drought stress it did not increase significantly; the cumulative amount of proline was low, only significantly increased in severe salt stress and severe drought stress conditions.

## 5. Conclusion

In summary, research on the relationships between plants and their environmental conditions has become a hotspot in present ecology studies. In this study, the growth and physiobiochemical characteristics of* T. chinensis* is able to adapt to the stress environment by adjusting the growth pattern and alternating soluble sugar and proline content, protective enzyme activity, and other physiobiochemical indexes to improve the ability to adapt to adversity.* T. chinensis *showed strong drought resistance and salt tolerance, and its growth conditions and physiobiochemical characteristics were related closely to the outside environmental factors, such as soil salinity and soil moisture status. On the other hand, the intercross compatibility of* T. chinensis *to salt and drought stresses can enhance the salt resistance under appropriate drought stress, and the dominant factor influencing the physiological biochemical characteristics of* T. chinensis* shows some differences with the changing of salt-drought intercross stress gradients. As* Tamarix chinensis* communities degrades in the Yellow River Delta, due to environment change and anthropogenic activities, then, the results of this study has certain theoretical and practical significance for the evaluation of* T. chinensis* resistance and reproductive technology, but the internal adjustment mechanism and adaptation mechanism need to be further researched in future.

## Figures and Tables

**Table 1:** Effects of* Tamarix chinensis* growth and biomass conditions with different salt and drought stresses treatment.

Treatment		Plant height (cm)	Main root length (cm)	Stem basal diameter (cm)	Overground biomass (g)	Underground biomass (g)
Water condition	Salinity content					
High soil moisture (mild drought)	CK	85.93 ± 7.65^b^	14.54 ± 1.25^a^	0.51 ± 0.12^a^	13.12 ± 2.89^b^	5.01 ± 0.97^b^
	0.4%	115.76 ± 10.28^a^	15.58 ± 1.67^a^	0.63 ± 0.26^a^	18.01 ± 3.35^a^	9.52 ± 2.71^a^
	1.2%	87.29 ± 5.96^b^	11.56 ± 1.87^b^	0.37 ± 0.14^b^	9.04 ± 3.02^c^	4.13 ± 1.85^b^
	2.5%	—	—	—	—	—

Low soil moisture (severe drought)	CK	85.70 ± 5.37^b^	11.51 ± 1.12^b^	0.47 ± 0.14^a^	12.89 ± 1.18^b^	4.86 ± 0.92^b^
	0.4%	98.58 ± 4.72^a^	12.57 ± 1.25^b^	0.49 ± 0.22^a^	15.67 ± 1.37^a^	7.03 ± 1.15^a^
	1.2%	74.43 ± 5.01^c^	15.68 ± 1.67^a^	0.35 ± 0.09^b^	6.95 ± 1.86^c^	3.72 ± 0.89^b^
	2.5%	86.24 ± 3.97^b^	10.89 ± 1.02^b^	0.36 ± 0.08^b^	5.26 ± 0.98^c^	2.63 ± 0.76^c^

The different letters within the same row indicate the significant difference at 0.05 level. “—”stands for plant individual death.
